# Photobiomodulation of the Visual System and Human Health

**DOI:** 10.3390/ijms21218020

**Published:** 2020-10-28

**Authors:** John Buch, Billy Hammond

**Affiliations:** 1 Johnson & Johnson Vision, Research & Development, Jacksonville, FL 32256, USA; 2 Department of Psychology, University of Georgia, Athens, GA 30602, USA; bhammond@uga.edu

**Keywords:** electromagnetic radiation, photobiomodulation, action spectrum, phototoxicity, visual system

## Abstract

Humans express an expansive and detailed response to wavelength differences within the electromagnetic (EM) spectrum. This is most clearly manifest, and most studied, with respect to a relatively small range of electromagnetic radiation that includes the visible wavelengths with abutting ultraviolet and infrared, and mostly with respect to the visual system. Many aspects of our biology, however, respond to wavelength differences over a wide range of the EM spectrum. Further, humans are now exposed to a variety of modern lighting situations that has, effectively, increased our exposure to wavelengths that were once likely minimal (e.g., “blue” light from devices at night). This paper reviews some of those biological effects with a focus on visual function and to a lesser extent, other body systems.

## 1. Introduction

James Maxwell, building on postulates from Gauss, Riemann, and Faraday, proposed in 1864 that an electromagnetic “disturbance” exists that travels in free space with the velocity of light [1]. His theory was later confirmed by Hertz around 1888 [1] and thus began our understanding of electromagnetic (EM) radiation. It is now accepted that EM radiation is the flow of photons that behave as both particles and waves, and is divided into bands depending on the propagating wavelength. To illustrate, gamma waves have wavelengths measured at the atomic level (<100 pm), while the wavelengths of radio waves are measured in miles. Despite a relatively long history of research, the effects of EM radiations on human biology are relatively understudied, and carries the oft-quoted paradox of being both necessary and antithetical to life. From this general aphorism, however, many specific questions can be posed: What are the common sources of EM? What wavelengths promote health, and which detract, and at what intensities? What and how are biological processes affected with acute and chronic EM exposure? These questions merit immediate comment.

The most commonly encountered source of EM radiation is the sun. Within the 48% of the sun’s radiation that actually reaches the earth’s surface [2], 49.4% is infrared (>700 nm), 42.3% is visible (400–700 nm), 6.3% is ultraviolet (UV) A (320–400 nm), 1.5% is UVB (290–320 nm), and 0.5% is UVC (<290 nm) [3]. The bulk of this paper will therefore concentrate on these wavelengths. However, the level of irradiance in the “visible” spectrum is much higher, owing to the evolutionary tuning of our visual system. That is, we see what is most energetic at the earth’s surface. The positive and negative effects of EM radiation on the eye will be discussed shortly.

Another commonly encountered source of EM radiation are radio frequency emitters. Radio frequency emitters have been around for some time (circa Heinrich Hertz, 1886) but not in such high numbers, nor have the detection devices for such emitters (cellular phones) been in such constant and close proximity to humans. As such, there has been concern that chronic exposure to this seemingly insignificant energy source (around 3 kHz–300 GHz) carries the potential for tissue damage. Reflecting such concerns [4], the National Toxicology Program published extensive results [5] on the effects aggregated over a lifespan of about 2 years, including in utero, of radiofrequency exposures on the brains and hearts of rats and mice. The study found significant pathological changes, such as DNA damage, using similar frequencies as those used by the telecommunications industry (900 and 1900 MHz), but the study used much higher energy exposures. Mice in the study, for instance, were exposed to intensities of up to 10 Watts/Kg (analogous to ~500 Watts for a 110-pound human) for around 9 h per day. Cell phone energy varies from about 2 Watts to around 0.2 Watts during a call. The relatively recent release of 5G cellular networks has its own health controversies [6,7], and of course, some individuals keep their cell phones within close proximity constantly.

Hence the conundrum when considering the interaction of EM radiation and biology: how are the relatively intense, acute, and easily quantifiable exposures used in animal models comparable to the relatively weak exposures that humans experience over a lifetime? Human behavior is complex. How does one link any complex outcome to any single (often weak) input with dozens of significant and confounding correlates? Not surprisingly, for example, epidemiological studies on the relation of cancer to cell phone usage are inconsistent at best [5].

Similar challenges are encountered when relating light damage to chronic ocular disease. Intense acute studies on animal models tend to show very clear results with easily described underlying mechanisms (for example, Barker et al. studied the protective effects of xanthophylls on short-wave light-induced retinal damage [8]). Long-term chronic studies tend to be inconsistent [9] and depend strongly on the nature of the disease. With respect to the latter, for instance, easily quantified (e.g., eyelid malignancies) or relatively acute conditions (e.g., photokeratitis) show a much more straightforward relation to actinic light than more difficult to quantify retinal diseases such as macular degeneration [10].

As a consequence, some researchers have decided to focus less on chronic degenerative diseases, which are characterized by long latencies and complex etiologies, and more on acute biological effects. If a stressor causes effect X, this is both meaningful in its own right and likely has implications for long-term disease. For example, cells phones (radio frequency detectors) are often carried around the waist and reproductive germ cells divide rapidly (spermatogenesis/oogenesis), which are easy to quantify in terms of cell counts and dynamics. There is good evidence that mobile phone exposure influences oxidative stress, sperm mobility, and fertilization [11]. Implantation of a UV-transmitting intraocular implant in one eye and a UV-blocking implant in the other causes loss of S-cone sensitivity in the UV-transmitting eye over a span of five years [12], not the same as AMD but S-cone loss is significant in its own right, a likely good indicator of approaching retinal disease [13].

Taking such a direct approach—light exposure vs biological response—allows a number of answerable questions to be posed. The first is methodological: How does one relate wavelengths, throughout the EM spectrum, to human biological response? This methodology was perhaps best worked out by plant photobiologists in the form of action spectra. Action spectroscopy is characterized by the measurement of biological effects that occur as a function of wavelength [14]. The Commission Internationale de l’Eclairage (CIE) defines the photobiologically active range from UVC (100 nm) to IRC (1,000,000 nm) [15]. The origin of this technology began with the obvious and differential light sensitivity of plants. It was used, for example, to originally identify chlorophyll as the pigment most likely involved in photosynthesis [16]. Photochemistry, in general, was based on the observation that reactants respond (react) to some wavelengths more strongly than others. It was natural to generalize these observations to the photobiology of organisms. At first, the emphasis was on damage: some wavelengths were more damaging than others [17]. After, studies shifted to the efficacy of certain wavelengths in mediating a variety of biological reactions. The effects were ubiquitous.

In this review, we identify and describe a number of these biological effects, most dealing with our most EM-responsive system (vision), focusing mainly on EM effects as quantified by action spectra. We also highlight the effects that EM radiation has on other body systems, thus attempting to collect this information into one reference.

## 2. Spectral Band Terminology

Summarizing the effects of wavelength is not straightforward, given the non-uniformity in defining various spectral bands across various disciplines. The boundaries of what is termed visible light are often defined with a low and high spectral end between 360 and 400 nm and 760–830 nm, respectively [18], although younger individuals can consistently see UVB, ca. 315 nm [19] and infrared, ca. 1100 nm under some circumstances [20]. How EM radiation passes through materials such as glass or quartz, water and ozone, or is absorbed or transmitted by proteins and water, can have different meanings for disciplines such as optical engineering, meteorological optics, or photobiology, respectively [21]. Photodermatology divides UVA/UVB at 320 nm, although CIE recommends 315 nm [22].

There are small discrepancies even within different ISO standards depending on the application. For example, ISO 21348 provides a process for determining solar irradiance and defines UVA as 315–400 nm, while ISO 8980 provides transmittance specifications for ophthalmic lenses and defines UVA as 315–380 nm. ISO 21348 defines “soft x-rays” as 0.1–10 nm, while ISO 20473 (optics and photonics) defines “extreme ultraviolet” as 1–100 nm. Superficial discrepancies can also be found within the same standard, such as ISO 21348, stating that the color purple begins at 360 nm while the visible light spectrum begins at 380 nm.

The commonly described “Blue Light Hazard” function is even a misnomer since over half of the rays that constitute this spectral range would be considered purple or violet in color. ISO 8980-3 defines the Blue Light Hazard range as 380–500 nm. The Blue Light Hazard can be further refined (e.g., 380–460 nm) based on the wavelengths of greatest potential risk (ISO/TR 20772). The term High-Energy Visible (HEV) light is sometimes used in lieu of the blue light hazard but tends to refer to the same wavebands. HEV ranges fluctuate from 380 to 500 nm [23], 400–500 nm [24], and others stating 430–510 nm [25] depending on the author’s particular field. This is different from the “Retinal Hazard Region” often defined as 400–1400 nm [26].

Consistent terminology across fields is important since light influences nearly every system of the body, and those effects are largely wavelength-dependent. Light, for instance, entrains circadian rhythms, for humans and most other species, ranging from cyanobacteria to “higher” animals (studied by chronobiologists). We have “clocks” throughout our bodies, most entrained by the master clock in the brain (the suprachiasmatic nucleus of the hypothalamus). The biochemical bases for such clocks are hormones such as melatonin, and the action spectrum for these mediators has been well established [27]. Light is also considered an essential therapy for seasonal affective disorder and is believed to be more efficacious when it contains a significant amount of blue (468 nm) light [28].

## 3. Introduction to EM Biological Effects

Since the original invention of lasers (essentially monochromatic light that could be more easily delivered), light has been used as a means of therapy. Specific wavelengths of light have been shown to be effective at reducing pain, inflammation/edema, and promoting the healing of wounds [29]. Light has been shown to be directly or indirectly associated with alertness, performance, and mood [30]. It can be used to stimulate hair growth [31] when hair is thinning (max = 830 nm) and to stimulate the production of collagen by the skin. Indeed, all cells contain light-responsive substrates (chromophores like cytochrome c oxidase) whose activity is altered by exposure to select wavebands of light. For example, mitochondria create energy for the cell and light can enhance that process (ATP production), changing transcription factors and the regulation of reactive oxygen species [32]. Blue light (400–500 nm), with or without red light (>600 nm), has been used for the treatment of acne [33].

**Figure 1:**
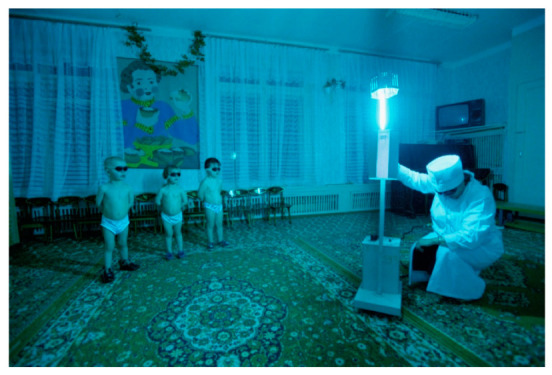
In sunless Lovozero, Russia, children bathe in ultraviolet light to produce vitamin D for their bones. Photo © Joe McNally.

Sunlight can also be used to synthesize (from cholecalciferol, cholesterol in the skin) an essential nutrient, Vitamin D (λmax = 300 nm, [34]). Simulated UV-exposure has been used in sunlight-deprived regions of the world (Figure 1). Vitamin D itself is the precursor to the steroid hormone calcitriol. Calcitriol regulates a number of cellular pathways known to be central to cancer progression and risk [35]. Vitamin D was originally added to milk in order to reduce rickets (weakened bone especially obvious with weight-bearing bones like the femur, which caused kids to become bow-legged) that occurred especially in Black children in inner cities, where buildings block the sun [36].

Each of these systemic effects has its own specific wavelength dependency. For example, cell culture studies have shown that maximum cell proliferation of fibroblasts occurs with light from 665 to 675 nm, whereas slightly longer wavelengths (810 nm) inhibit cell division [37]. The field of biophotonics uses specific wavelengths of light to achieve imaging down to the cellular level [38]. It is sometimes the case that even very small changes in wavelength can create large changes in biological response at the cellular level. Moon et al. exposed retinal pigment epithelial cells loaded with A2E to 449, 458, and 470 nm light at varying time intervals [39]. Despite the equal energy of the exposure, small changes in wavelength resulted in large changes in cell viability. For example, after 48 h of exposure, the production of reactive oxygen species was three times higher for cells exposed to 449 vs 470 nm light. Most recently, long wave light (670 nm) has been used to stimulate mitochondrial function in the retina as a means of retarding age-related decline in photoreceptor function [40].

## 4. Phototoxicity, Dosimetry, and Action Spectrum of the Visual System

Light is the primary stimulus used by the visual system. What is less obvious is that different wavelengths of light influence literally every aspect of the visual process. The early philosophers, from the Greeks to the Chinese, focused mostly on how wavelength related to the perception of color. The first systematic/empirical study of wavelength and color has been attributed to Isaac Newton (Opticks, 1704), but, as art and science flourished hand in hand through the Renaissance, so did interest in the underlying optics of color. Prominent intellectuals like Goethe published treatises such as Zur Farbenlehre (‘Theory of Color,’ 1810). It was not until 1916 [41], however, that human spectral sensitivity curves were published and then codified by the CIE [42]. What followed, and is beyond the scope of this review, were extensive studies of how the optics of the eye are affected by wavelength [43], as well as characteristics as diverse as spatial [44] and temporal vision [45]. Along with this work came the realization that different wavelengths of light are more or less damaging to the visual system, and other surface tissues such as skin. Van Norren and Vos published a comprehensive review of the history of research on actinic action spectra [46]. This empirical work mostly began with animal models (the work of Noell and Ham in the 1960s) but the damaging effects of light were also being actively considered in the ophthalmic literature [17].

Light, in this case, with its potential to harm, refers mostly to the higher energy portions of the EM spectrum, although significant exposure to lower energy light (say infrared) can also predispose an individual to damage. For example, thermal lensing can defocus NIR at high intensities and increase susceptibility [47]. Historically, the primary source for high-energy light came from the sun. Even now, although many common devices (mobile phones, computers, etc.) emit light in the short-wave portions of the spectrum, the amount is likely not significant enough to initiate photo-oxidative damage [48].

Light that damages the eye is termed phototoxic. Phototoxicity can take different forms depending on the wavelength and intensity of the approaching EM radiation. Ocular phototoxicity is subdivided into photothermal (photocoagulation), photomechanical (photoacoustic), or photochemical damage. Photothermal damage typically occurs with the longer visible and NIR wavelengths of 600–1400 nm, where photons have enough energy to cause molecular vibration without splitting electrons from atoms within the molecule. This is inferred as heat and is particularly damaging to the crystalline lens (e.g., “glassblowers” cataract) [49] and intentionally applied to the retina for laser photocoagulation of certain disease states (e.g., diabetic retinopathy, retinal edema, etc.) [26]. Photomechanical damage generally refers to a very short pulse (e.g., nanoseconds) of very high energy (e.g., megawatts/cm^2^) that results in a thermoelastic pressure wave [50]. This shock wave results in microcavitation bubbles and permanent tissue change. A common example is the neodymium-doped yttrium aluminum garnet (Nd:Yag) laser used to punch a hole in the peripheral iris (iridotomy) to help control glaucoma. The Nd:Yag laser is often set to 532 nm, 700–900 mW, for 0.1 s [51]. Photochemical damage is thought to occur when photons are absorbed by the molecules of a tissue, exciting the electrons from the ground to excited state (i.e., the formation of free radicals or reactive oxygen species). This is typically associated with long exposure times and higher energy light [26]. Photochemical ocular damage is associated with the pathogenesis of diseases such as macular degeneration and choroidal neovascularization. Photochemical exposures are additive according to the Bunsen-Roscoe law of photochemistry which basically states that damage can occur from an intense radiation source for a short time, or a less intense source for a longer period of time [52]. Paradoxically, the damaging photochemical process is also utilized in photodynamic therapy (PDT) where unwanted anomalies are treated with a photosensitizing drug then exposed to EM radiation (e.g., 689 nm) to induce the free radical process and reduce the anomaly [53].

There are many factors to consider when calculating the dose of when a particular wavelength can cause harm to a tissue. Apart from the numerous environmental factors [54,55], radiation that reaches the ocular surface is also dependent upon anatomical factors that might limit its entry into the eye [56,57] such as the size and shape of the nose, skin reflectance, protruding eye (exophthalmos), sunken eye (enophthalmos), eyelid droop (ptosis), eyelashes, eyebrow, and prominence of the supraorbital ridge. Once the radiation reaches the ocular surface, then factors such as wavelength (action spectra), aversion responses (looking away, pupil constriction), tissue repair, size of the light source, exposure intensity, exposure area, and exposure duration all play a role [52]. Nonetheless, general exposure guidelines are provided in Tables 1–5 where appropriate.

Several recent reviews are available that discuss the impact that electromagnetic radiation has on the eye [46,58], skin [59,60], and other biological functions [61]. In brief, as noted, UV damage is photochemical, not photothermal. “Actinic ultraviolet” is most significant below 320 nm (UVC and UVB) but affects deeper structures of the eye based on penetration (e.g., UVA penetrates deeper and may be more significant for retina). More recent reports suggest that UVB [62] and near UVA (360–400 nm) [63] are important for myopia control. Furthermore, blue (464 nm) and red (634 nm) may be important for emmetropization when coupled with a multifocal lens [64].

Although, significant exposure to lower energy light (say infrared) can also predispose an individual to damage. For example, thermal lensing can defocus NIR at high intensities and increase susceptibility [47].

### 4.1. The Eyelids

Table 1 summarizes the effects of high-energy light on the eyelids. As shown in the table, most of the effects of light on the eyelids can be predicted by the general effects of light on skin, with the exception that the skin of eyelids is thin (as light filters go, they have an average optical density of around 2.1 ± 0.3 in the visible range, [65]). They are also somewhat protected by the supraorbital bone and eyebrows [58]. Lids, ridges, and brows combine to also provide some protection to the cornea, the other major ocular surface tissue. The action spectra for the cornea is summarized in Table 2.

### 4.2. The Cornea

As shown in Table 2, many of the significant effects to the cornea are highly wavelength-dependent and in the low UV range. As ocular structures go, the cornea represents the main interface between the eye and environment, and hence absorbs the largest amount of radiation [80]. This, perhaps, saves deeper structures to a large degree but creates great susceptibility of the cornea to very low wavelengths. This is a risk both indoors and out. With respect to the former, UV light is used in entertainment such as black lights at nightclubs, and mass presentations of photokeratitis have been reported [82]. UV used as a disinfectant has been reported in 41 cases of photokeratitis in poultry workers [83]. UV promotes inflammation (keratitis) though a number of interleukins, cytokines, and matrix metalloproteinases that, ultimately, mediate cell damage [80]. It should be noted that corneal crosslinking is a procedure used to stiffen an ectatic cornea by the combination of a photoinducer (vitamin B2) and 370 nm light [84]. The light used to conduct the corneal crosslinking procedure (365 or 370 nm) can also be used to effectively treat some cases of infectious keratitis [85].

### 4.3. The Conjunctiva

The other ocular surface tissue that is also an eye-environment interface is the membranous conjunctiva. Effects of EM radiation on the conjunctiva are outlined in Table 3. The most obvious clinical manifestations of light damage to the conjunctiva are pterygium and pinguecula (noncancerous growths, often nasal, visible on the sclera). Since the conjunctiva interfaces with the external environment, many factors can induce general inflammation of the conjunctiva (conjunctivitis). This has made recent news, for example, as a relatively unique symptom of COVID-19. Early in the natural and recent history of the novel coronavirus pandemic was the observation that the condition can cause changes (often irritation) to the conjunctiva (indeed, the first doctor who identified the new condition was an ophthalmologist, Li Wenliang). These changes manifest as dry eye, blurred vision, and mild follicular conjunctivitis [86]. Ocular changes, like conjunctivitis, do not typically accompany many other systemic infections (like influenza or even other coronaviruses like SARS or MERS). Almost 80% of conjunctivitis cases are specific to the adenovirus [87] and occur early in life (0–4 years of age). Reaction of the eye to systemic infection is relatively rare. This implies that CoV may be relatively unique in its ability to influence surface tissues of the eye. This may be why this strain of coronavirus (HCoV-NL63) caused alarm when identified initially in an infant displaying conjunctivitis. A subsequent study of 28 cases of children with confirmed HCoV-NL63 infections showed that 17% had conjunctivitis [88]. Although the disease could come in contact with the conjunctiva as an aerosol, the presence of CoV in the conjunctival sac [89] suggested it might also arise from within the patient and be expressing through lacrimal fluid. Viruses often absorb in the UVC range [90] which is why UV light is sometimes used as a germicidal (e.g., hospital rooms sometimes use upper room germicidal irradiation) and is being actively considered to help retard the spread of CoV, and UVC appears most effective [91].

Almost 80% of conjunctivitis cases are specific to the adenovirus [87] and occur early in life (0–4 years of age). The reaction of the eye to systemic infection is relatively rare. This implies that CoV may be relatively unique in its ability to influence surface tissues of the eye.

### 4.4. The Iris and Crystalline Lens

Light passing through the cornea/conjunctiva (which now has removed much of the UV) passes through the pupil but is also incident on the pigmented iris. The iris is contiguous with the retinal pigment epithelium (to wit, a darker iris reflects a darker posterior fundus) and it seems clear that the degree of pigmentation (unlike skin, iris pigmentation stays relatively constant) evolved to screen the further penetration of high-energy light [99]. This is why light iris color (reduced filtering) is thought to be a significant risk for uveal melanoma [100]. In contrast, darker irises (absorbing more light energy), are linked to a higher risk of cataract. Cumming et al. posited that the absorbance of thermal energy by a darker iris could raise the energy state of the crystalline lens and predispose it to other forms of photo-oxidative damage [101]. Action spectra for the crystalline lens is provided in Table 4.

### 4.5. The Aqueous Humor and Vitreous Humor

UVB radiation is absorbed by the cornea, aqueous humor, and crystalline lens, with <1% reaching the retina [111]. Although as recently noted by Hammond, there is large (over a factor of 30) individual variation in the quantity of UVB reaching the retina prior to the age of about 30 years [19]. The remaining UVB is not significantly absorbed by the aqueous and vitreous humors, although thermal effects are possible [112]. UVC is also mostly absorbed by the anterior media but the small percent remaining likely does influence the aqueous humor. The antioxidant, ascorbic acid (water-soluble Vitamin C), is present in aqueous humor and has a peak absorbance in the UVC (around 265 nm [113]; also, proteins and amino acids in the aqueous absorb in the UVC). In various species, including humans, the presence of a cataract is correlated to decreased ascorbic acid within the aqueous humor [114]. A recent study documented a significant decrease in rabbit ascorbic acid concentrations after acute UV exposure [115]. This UV-induced reduction of aqueous humor ascorbic acid levels was minimized in eyes with UV-absorbing contact lenses [111]. Lledo et al. recently reported that yellow filters can significantly increase the amount of melatonin in the anterior chamber of the eye. This, in turn, reduces the production of aqueous humor, presumably by inhibition of the non-pigmented epithelium of the ciliary body, and reduced intraocular pressure approximately 40% in rabbits [116]. Light effects on vitreous are also apparent. An increase in vitreous catalase activity by 33% was shown in calf eyes irradiated with UVA for 3 h [117]. As with most ocular structures, effects on vitreous are wavelength-dependent. X-rays appear to have no effect on the vitreous [118] but IRA (ca 780–1400 nm) can cause a blanched retinal lesion or edema proceeding to vitreous hemorrhage [71].

### 4.6. The Retina

Table 5 provides a summary of the action spectra for the retina. The retina is unique from the other tissue types in that it contains cells that do not undergo mitosis (hence, damage is often irreversible). It is also the highest metabolically active tissue in the body. This metabolic activity results in excessively high oxygen tension, combined with high lipid content, and photosynthesizers (those that are always there, like rhodopsin, and others that increase with age, like A2E) makes for an exceptionally vulnerable tissue (retinal degeneration is the most common form of blindness in developed countries).

Although, as recently noted by Hammond et al., there is large (over a factor of 30) individual variation in the quantity of UVB reaching the retina prior to the age of about 30 years [19].

Atmospheric oxygen, in its ground-state or triplet form (3 O2), is a diradical, meaning that it possesses two unpaired electrons that spin in parallel orbits. This coordination of electron orbits tends to stabilize the triplet form of oxygen. If triplet oxygen, however, absorbs enough light energy to reverse the spin of one of its unpaired electron orbits, it converts to a more reactive singlet form (1 O2). The singlet form of oxygen stays reactive for a relatively long time period since conversion back to the triplet form is spin-forbidden. Receptoral outer segments contain high quantities of polyunsaturated fatty acids (PUFA). Reactive oxygen species can abstract hydrogen atoms from PUFA-rich receptoral membranes, ultimately leading to peroxidation of the lipid. The withdrawal of hydrogen will convert the PUFA into an organic radical that may then react in a similar manner with adjacent PUFA molecules. This chain sequence, left unquenched, slowly begins the formation of the lipid-based oxidation products that populate the older retinal pigment epithelium (the RPE does not have the enzymes to break down oxidized lipids, so they simply accumulate). This basic model [134] is a common one used to describe the natural history of acquired retinal degeneration, often combined with other diatheses such as genetic susceptibility, impairment in choroidal circulation, and thickening of Bruch’s membrane.

### 4.7. Other Ophthalmic-Related Effects

Finally, it is clear that wavelength can differentially influence the visual system as a whole. Table 6 is a sample of such conditions, largely deleterious, where wavelength differences are known to have a meaningful effect. For example, Revell showed that exposure to 420 nm light had a significant effect on increasing the alertness of young male subjects, whereas the same exposure to 600 nm had the opposite effect [135].

## 5. Photobiomodulation Effects on Human Health

In 2015, Juanita Anders (President of the American Society for Laser Medicine and Surgery) and colleagues argued for the acceptance of the specific term “photobiomodulation,” as opposed to say, low-level light/laser therapy, for the use of non-ionizing EM radiation (mostly visible and infrared) as adjuvant clinical therapy (e.g., wound healing, anti-inflammatory) [144]. Recently, the term “photobiomics” has been introduced to recognize the effects of light therapy on the microbiome [145]. The ideas behind this type of photomedicine (using light for clinical benefit), however, were introduced far earlier. In the late 1960s, for example, Endre Mester, who was testing the effects of lasers as carcinogens, discovered that infrared lasers, instead, stimulated hair growth in mice [146]. There are now about 50 laser-based devices on the market for the purpose of stimulating hair growth in humans—reviewed by Fayne et al. [147].

Applications of photobiomodulation (PBM), however, extend throughout medicine. For example, NIR treatment of the heart is being considered for patients with acute myocardial infarction and ischemia to promote revascularization, so-called cardio-light [148]. Transcranial NIR is being investigated as a treatment for a number of psychiatric conditions such as anxiety disorders [149]. It has been known for some time that a fraction of long-wave light delivered through the skull can reach neurons (hence, the success of techniques such as optical neuroimaging or optogenetics) and moderate their activity. Indeed, although in inchoate stages, there is hope that transcranial PBM may be an effective treatment for Alzheimer’s disease and other dementias [150] and degenerative movement disorders such as Parkinson’s [151]. A “photoceutical” approach has been used in wound care for years [152] with clear evidence of its efficacy [153]. Longer-wave light tends to penetrate tissues and is absorbed by enzymes like cytochrome c oxidase which can activate mitochondria. Markowitz et al. recently used PBM therapy to treat patients with the dry (more common and difficult to treat) form of macular degeneration. They found that application of multiple wavelengths (590, 660, 850 nm) over a year, compared to a sham treatment, significantly reduced the volume of central drusen and improved visual function [154].

Although most of the work in this area has focused on long-wave and near-infrared light, short-and mid-wave light may have therapeutic potential as well (e.g., modulating opsin signaling [155]). This has long been obvious for issues such as jaundice in babies (e.g., blue light therapy breaks up bilirubin, [156]) or for more utilitarian purposes such as curing of dental compound [157]. In recent years, however, the applications have expanded. For example, Magni et al. has shown that short-wave light (ca. 420 nm) reduces the metabolism (and hence proliferation) of fibroblasts which could reduce keloid scarring [158]. Mid-wave (green) light, introduced with implantable fiber optics, may be optimal for bone regeneration [159]. Light in the range of 350–450 nm is known to photoreactivate the harmful effects of far UV (200–300 nm) in some organisms. Interestingly, photoreactivation and circadian photoentrainment may have both evolved from the same short-wave photoreceptors as a means of DNA repair [160].

As noted throughout this review, different wavelengths prompt different effects. For example, “red” and NIR light increase activity within the TGF-β signaling pathway (regulates cell growth/differentiation), whereas “blue” light inhibits that same pathway [155]. Like alternating cold and hot compresses for edema, alternating wavelength to achieve specific biological effects may ultimately prove the most efficacious PBM approach for many conditions [161]. In addition, evidence is mounting that red and NIR (600–1070 nm) are helpful in stopping the neurodegeneration of some disease states such as Alzheimer’s and Parkinson’s [162].

## 6. Conclusions

Absorption spectroscopy is a technique that is used to measure wavelength absorption across the EM spectrum. It is often used to determine the identity of specific chemical components. The what, however, raises questions of the why, which can also often be inferred from the unique spectral characteristics of a material. Studying spectra provide insights into function. This is obvious in fields such as astronomy (e.g., cosmological redshift shows the universe is expanding not contracting). It is true also, however, of biology. Mitochondria and chloroplasts, both utilizing similar cytochromic enzymes, likely descended from the same ancient endosymbiosed bacteria [163]. Chlorophyll A and B have absorption peaks in the short-wave and long-wave visible spectra (each peak effecting electron excitation states differently). To photobiologists, the visible spectrum is therefore referred to as Photosynthetically Active Radiation (PAR) [163]. Mitochrondria are also differentially influenced by short- and long-wave light, albeit in opposition: For example, the absorption of short-wave light by the mitochondria of retinal ganglion cells increases ischemic cell death but it is decreased by exposure to long-wave visible light [164].

A specific, and often elegant interaction between wavelength and mechanism, tends to typify visual biology. Humans can discriminate millions of colors based on relatively subtle variations in retinal opsins (three amino acid substitutions account for the 30 nm difference between the absorption peak of mid- and long-wave cones [165]). Even passive filtering appears to be carefully tuned. The central cone photoreceptors can afford light loss and hence utilize pigments that (sometimes strongly) filter the bottom third of the visible spectrum (lutein and zeaxanthin in the inner retinal layers). In contrast, rod photoreceptors that are peripheral and adapted for high light sensitivity, take advantage of molecules like alpha-tocopherol that are transparent to visible light. Each receptor type is highly vulnerable to lipid peroxidation, but a different antioxidant system is used in each case.

The entire retina is screened by the natural crystalline lens which absorbs most of the UV [19] and strongly in the visible range up to around 440 nm. Marie et al. used A2E-loaded RPE cells with 10 nm stepped exposures across the visible spectrum to measure a precise action spectrum for this model of retinal damage [166]. The authors concluded that the waveband between 415 and 455 nm generated the largest magnitude of damage (reactive oxygen species and mitochondrial dysfunction).

If it is the case that human biology in general, and the visual system in specific, is very finely adapted to the EM spectrum, some questions have taken on recent urgency. What is the consequence of how modern technology is changing the landscape of our light exposure? What is the action spectrum of now common conditions like glare, ocular fatigue, and visual strain? Is the lack of natural light during development fueling a pandemic of myopia? Can light be used therapeutically in conditions ranging from mental state to wound healing?

Astrophysicists often define the habitability of exoplanets (the Goldilocks zone) based on EM radiation (e.g., too much UV prevents the formation of nucleic acids). Human biology, like all life on earth, evolved extreme sensitivity to even minor variation in wavelength exposure. Defining the exact nature of these effects across a range of biological effects is an important goal.

## Figures and Tables

**Table 1:** Electromagnetic radiation action spectra and dosimetry thresholds for the lids.

Wavelength	EMR Phototoxicity and Dosimetry of the Lids	
	Effect	Reference
UVB 280 < 315 nm	Average erythemal UV dose of Americans is about 25,000 J/m^2^/year.	[66]
	3X more carcinogenic than UVA (e.g., squamous cell carcinoma, SCC).	[66]
	Squamous cell carcinomas account for about 8%, and melanomas about 2%, of all-cause cancer in Croatia.	[67]
	Basal cell carcinomas (BCC) account for 80–90% of all malignant tumors of the eyelid.	[68]
	Excess exposure increases risk of erythema, melanogeneisis, DNA damage, immune suppression, photo-aging.	[66]
	UVB is major cause of sunburn, which is leading risk factor for melanoma and non-melanoma skin cancers.	[66]
	BCC may be due to strong UVB exposure at a young age, whereas SCC appears due to chronic/cumulative exposure.	[10]
UVA 315 < 400 nm	Photoaging: main effect on the lids is sagging skin.	[66]
Visible 380 < 760 nm	Intense visible (e.g., 532 nm laser pointer) has been shown to cause ecchymosis of upper and lower eyelids.	[69]
Infrared	Photodynamic therapy (at 634 nm) is an effective treatment for basal cell carcinoma.	[70]
	Thermal radiation can cause burns ranging from mild to third degree and eventually skin death.	[71]
Exposure Limits (see text discussion)	UVR 3 mJ/cm^2^ for 8 h daily to avoid redness (erythemal), and this is one-third to one-quarter of the minimal erythemal dose.	[72]

**Table 2:** Electromagnetic radiation action spectra and dosimetry thresholds for the cornea.

Wavelength	EMR Phototoxicity and Dosimetry of the Cornea	
	Effect	Reference
UVC 100 < 280 nm	Momentary exposure to UVC can cause photokeratitis such as welders flash.	[73]
	Susceptibility to photokeratitis may peak at 270 nm with exposure thresholds as low as 3 mJ/cm^2^.	[74]
UVB 280 < 315 nm	10 h of UVA or 23 min of UVB can cause photokeratitis.	[75]
	UVB absorption is 1.8 times higher in the anterior 100 nm of the human cornea than in the posterior layers. The absorption coefficients of the epithelium and Bowman’s membrane are higher than the stroma, but the stroma absorbs more due to thickness. Tryptophan and ascorbic acid absorb UVB.	[76]
	300 nm causes apoptosis in all three layers of the cornea and induces keratitis. Apoptosis in all layers of the cornea occurs 5 h after exposure.	[77]
	UVB light can accelerate the physiological loss of corneal epithelium be two mechanisms, shedding and apoptosis.	[10]
	The biological damage potential at 295 nm is 375 times more than the biological damage potential at 320 nm.	[78]
UVA 315 < 400 nm	Climatic droplet keratopathy—chronic UVA and UVB.	[10]
	Corneal crosslinking: primary treatment for corneal ectatic disease, involves application of vitamin B2 (riboflavin) + 370 nm to stiffen the cornea.	[79]
	Epithelium: pseudo-keratinization, polyhedral intermediate cells, necrosis, lymphatic infiltration.	[80]
	Bowman’s membrane: detachment from epithelium, thickened, micro-bleedings.	[80]
	Stroma: swelling and collagen disorganization, inflammatory cells, angiogenesis blood vessels.	[80]
	Endothelial detachment.	[80]
Visible 380 < 760 nm	Punctate keratitis caused by 532 nm laser pointer.	[69]
	Climatic droplet keratopathy with higher blue (400–500 nm) light exposures, in addition to UVA and UVB.	[81]
NIR 760 < 1400 nm	The cornea transmits 96% of incident infrared in the 700–1400 nm range, limiting sensitivity to IR harm, especially in the 750–990 nm range. Significant exposure results in causing protein coagulation which can cause irreversible damage especially on endothelium layer. High-dose IR damage to the cornea causes immediate pain and vascularization, with potential of loss of transparency and opacification in response to burns that causes ulcers.	[71]
Exposure Limits (see text discussion)	315–400 nm: 1 J/cm^2^ for exposure time < 1000 s 1 mW/cm^2^ for time ≥ 1000 s 180–400 nm: 3 mJ/cm^2^ pulsed hazard 770–3000 nm 1.8t^−0.75^ W/cm^2^ for time < 20 s 0.1 W/cm^2^ for time > 20 s 1.8t^0.25^ J/cm^2^ for time < 45 s	[52]

**Table 3:** Electromagnetic radiation action spectra and dosimetry thresholds for the conjunctiva.

Wavelength	EMR Phototoxicity and Dosimetry of the Conjunctiva	
	Effect	Reference
UVC 200–280 nm	Erythema: Although only 1% of 254 nm may penetrate the stratum corneum, mild erythema still results.	[74]
UVB 280–315 nm	Ocular surface squamous neoplasia (OSSN) declines by 49% for each 10 degree increase in latitude.	[92]
UVB, UVA and Visible	Pterygium: hyperplasia of the bulbar conjunctiva that grows over the cornea.	[10]
	Pterygium: Associated with long-term exposure to UVA and UVB.	[10]
	Pterygium: High prevalence between latitudes ± 37 degrees.	[93]
	Pterygium may initiate by UV-induced changes in corneal epithelial stem cells.	[94]
	Pinguecula: fibro-fatty degenerative change in bulbar conjunctiva.	[10]
	Pinguecula: Weak association with long-term UVA and UVB.	[10]
	Pinguecula: May be a histological link with sun-induced skin changes.	[95]
	448 nm at 0.8 mW/cm for 6 h resulted in lysosomal membrane permeabilization of the conjunctiva.	[96]
	Conjunctival UV autofluorescence can be used to accurately determine the time spent outdoors.	[97]
	Damage of conjunctiva caused by 532 nm laser pointer.	[69]
Exposure Limits (see text discussion)	270 nm: 3 mJ/cm^2^ within minutes can cause conjunctival injection, chemosis, damaged epithelial cells, and the presence of inflammatory cells.	[98]

**Table 4:** Electromagnetic radiation action spectra and dosimetry thresholds for the crystalline lens.

Wavelength	EMR Phototoxicity and Dosimetry of the Crystalline Lens	
	Phototoxic Effect	Reference
UVB 280 < 315 nm	295–325 nm associated with crystalline lens damage and cataract formation. Cortical and posterior subcapsular cataracts associated with intense 295–325 nm exposure delivered over days.	[72,102]
	315 nm contributes significantly to cataract formation	[102]
	Risk for cortical cataracts increased 1.6-fold when the cumulative UVB exposure doubled. No association of nuclear cataracts and UVB, or between cataracts and UVA—Chesapeake Bay Study.	[103]
	Chronic UVB exposure linked to cortical cataract.	[72]
	Variations in individual behavior can be a reason for up to a 18-fold difference in UVB exposure.	[104]
	280–315 nm is the most biologically active EM radiation band.	[74]
	Men with higher levels of average annual UVB were 1.36x more likely to have more severe cortical opacities than men with lower levels.	[105]
UVA 315 < 400 nm	If the radiant energy exposure was continuous and if no repair processes occurred, it would take 10 h of UVA to damage the cornea but 26 h to damage the lens. Lens damage due to UVB exposure would take 245 h, but the cornea would be damaged in 23 min.	[106]
	A generalization of the findings indicates that lens damage thresholds for UVA are in the J/cm^2^ range, and for UVB in the mJ/cm^2^ range.	[106]
	315–400 nm accelerates crystalline lens aging.	[102]
	325 nm at 260 J/cm^2^ created cataracts in rhesus monkeys.	[107]
Infrared	Cataracts induced in rats with 1090 nm, 197 W/cm^2^, multiple exposure times.	[108]
	Infrared causes lens changes in molecular weight and protein backbone structure, age-related types of cataract.	[109,110]
Exposure Limits (see text discussion)	315–400 nm: 1 J/cm^2^ for exposure time < 1000 s 1 mW/cm^2^ for time ≥ 1000 s 180–400 nm: 3 mJ/cm^2^ pulsed hazard 770–3000 nm 1.8t^−0.75^ W/cm^2^ for time < 20 s 0.1 W/cm^2^ for time > 20 s 1.8t^0.25^ J/cm^2^ for time < 45 s	[52]

**Table 5:** Electromagnetic radiation action spectra and dosimetry thresholds for the retina.

Wavelength	EMR Phototoxicity and Dosimetry of the Retina	
	Effect	Reference
UVC 200 < 280 nm	UVC causes time-dependent apoptosis of RPE cells.	[119]
UVB 280 < 315 nm	UVB energy from 0.2 to 0.4 J/cm^2^ induces decreased phagocytic activity of RPE cells.	[120]
UVA 315 < 400 nm	338 nm: Lipofuscin, a conglomerate of modified lipids and bisretinoids, is susceptible to photochemical changes leading to irreparable cellular damage.	[121]
	A2E, a lipofuscin fluorophore, has two peak absorbance rates, including one in the UVA range at 338 nm.	[121]
	325 nm can produce retinal lesions.	[122]
	315–400 nm: AMD linked to strong UVA exposure, mitochondrial DNA and RPE cells are particularly susceptible to 390–400 nm radiation within UVA, 380 nm was noted to cause damage to rat photoreceptor cells—particularly rods.	[123]
	Patients with macular degeneration have a higher rate of poor tanning ability and glare sensitivity.	[124]
	The retina was exquisitely susceptible to damage by UVA light, requiring irradiances 50–80 times lower to cause permanent photoreceptor cell damage with this wave-band compared to green light.	[125]
Visible 380 < 760 nm	Re-analysis of the Chesapeake Bay watermen study demonstrated an association between blue light exposure and AMD. Individuals with more sunlight exposure are at a significantly increased risk of AMD.	[81,126]
	400–480 induces photoreceptor cell death via apoptosis, consequently killing RPE cells.	[123]
	400–550 solar retinitis. Violet to blue light can cause temporary conditions such as ‘red vision’ (erythropsia) and reduction in night vision—especially in aphakic patients.	[72,102]
	400–550 are toxic to the aging retina as it loses antioxidant protection contributing to AMD.	[127]
	415–555 provided maximum loss of RPE.	[128]
	390–550 can irradiate lipofuscin, compromising lysosomal integrity and impairing activities of catalase, superoxide dismutase, and cathepsin while inducing lipid peroxidation—ultimately damaging mitochondrial DNA and RPE cells.	[123]
	Removal of the “blue” component of light significantly decreases retinal damage after high intensity exposure.	[129]
	Toxicity of “blue” led light and A2E is associated to mitochondrial dynamics impairment in ARPE-19 cells: implications for age-related macular degeneration.	[130]
	Short-wave light triggers cellular stress responses that may be involved in RPE disease development, which has implications for pathogenesis of AMD.	[131]
	Short-wave light increases production of reactive oxygen species (ROS), inducing oxidative stress and triggering photoreceptor cell and RPE death, which are risk factors for AMD.	[132]
	403 nm: regenerates rhodopsin.	[123]
	404 nm: cytochrome oxidase and RPE inhibited.	[123]
	430 nm: Maximum A2R excitation degrades RPE cells.	[123]
	432 nm: Damage to RPE cells via absorbance of all-trans-retinal.	[121]
	439 nm: Disrupt retina blood barrier in rats.	[123]
	447 nm: Peak A2E excitation rate harms RPE cells.	[121]
	448 nm: RPE disruption.	[96]
	460 nm: greater retinal damage by blue (460 nm) compared to green (530 nm) and red (620 nm) LEDs.	[133]
	470 nm: Damage to photoreceptor rods and RPE.	[123]
	488 nm: RPE disruption above ANSI’s photochemical MPE.	[121]
Infrared	Thermal damage causing enzymes to denature which contributes to permanent damage to the photoreceptor and RPE. Deep retinal coagulation, may involve choroid.	[71]
Exposure Limit (see text discussion)	305–700 nm 2 mW/(cm^2^ sr) for time > 10,000 s (phakic eye) White light source 0.22 mW/cm^2^ for time > 10,000 s 380–1400 nm 0.7 W/cm^2^ for time > 10 s and retinal image diameter > 1.7 mm	[52]

**Table 6:** The relation of wavelength to other ophthalmic-related issues.

Wavelength	Ophthalmic-Related Effect	Reference
UVA 315–400 nm	Blurred vision: Secondary to photokeratitis.	[136]
	Myopia development: 360–400 nm might be important for both preventing myopia progression and the onset of myopia.	[63]
Visible 380 < 760 nm	Migraine sensitivity: 447, 590, and 627 nm aggravates migraines, while 530 nm lessens severity.	[137]
	Glare via fluorescence: Near UV or short visible wavelengths induces a blue green fluorescence, which can be a source of intraocular veiling glare. Exposure to near UV/blue wavelength sources can influence a glare intense enough to reduce visual performance. Exposure to wavelengths longer than 365 nm induce weaker but progresses more towards red color, with 365 nm a lens absorption peak rate.	[138]
	Filtering 465–480 nm light may lead to a decrease in intraocular pressure by increasing melatonin levels in the anterior chamber.	[116]
	Chromatic aberration: 2.50 D difference within the eye between violet (360 nm) and red wavelengths (760 nm): may impair high-frequency contrast.	[139]
	Intrinsically photosensitive ganglion cells peak sensitivity at 480 nm.	[140]
	Blue haze /visual range: veiling due to short-wave dominant atmospheric haze limits how far an individual can see.	[141]
	High-intensity (blue or xenon) headlights: induce glare discomfort/disability particularly in older drivers.	[142]
	Photophobia/glare discomfort: shorter wavelengths are monotonically related to higher photophobic (greater squint) and aversion responses.	[143]
Infrared	Infrared may decrease discomfort.	[102]

## Abbreviations

A2E	N-Retinylidene-N-retinylethanolamine
AMD	Age-related macular degeneration
ANSI	American National Standards Institute
ATP	Adenosine triphosphate
BCC	Basal cell carcinoma
CIE	Commission Internationale de l’Eclairage
COVID	Coronavirus disease
DNA	Deoxyribonucleic acid
EM	Electromagnetic
EMR	Electromagnetic radiation
HEV	High-energy visible (light)
IR	Infrared
ISO	International Organization for Standardization
MERS	Middle East Respiratory Syndrome
MPE	Maximum permissible exposure
OSSN	Ocular surface squamous neoplasia
PAR	Photosynthetically active radiation
PBM	Photobiomodulation
PDT	Photodynamic therapy
POS	Reactive oxygen species
PUFA	Polyunsaturated fatty acids
RPE	Retinal pigment epithelium
SARS	Severe acute respiratory syndrome
SCC	Squamous cell carcinoma
UV	Ultraviolet
